# Leptin Is Associated with Testosterone, Nutritional Markers, and Vascular Muscular Dysfunction in Chronic Kidney Disease

**DOI:** 10.3390/ijms25147646

**Published:** 2024-07-12

**Authors:** Crina Claudia Rusu, Ina Kacso, Diana Moldovan, Alina Potra, Dacian Tirinescu, Maria Ticala, Remus Orasan, Cristian Budurea, Florin Anton, Ana Valea, Cosmina Ioana Bondor, Mara Carsote

**Affiliations:** 1 Department of Nephrology, University of Medicine and Pharmacy “Iuliu Hatieganu” Cluj, 8 Victor Babes, Street, 400012 Cluj-Napoca, Romania; 2 Department of Nephrology, County Emergency Clinical Hospital Cluj, 3-5 Clinicilor Street, 400006 Cluj-Napoca, Romania; 3 Nefromed Dialysis Center, 40 Ana Aslan Street, 400528 Cluj-Napoca, Romania; 4 Department of Cardiology, University of Medicine and Pharmacy “Iuliu Hatieganu” Cluj, 8 Victor Babes, Street, 400012 Cluj-Napoca, Romania; 5 Department of Endocrinology, University of Medicine and Pharmacy “Iuliu Hatieganu” Cluj, 8 Victor Babes, Street, 400012 Cluj-Napoca, Romania; 6 Department of Medical Informatics and Biostatistics, University of Medicine and Pharmacy “Iuliu Hatieganu” Cluj, 6 Pasteur Street, 400349 Cluj-Napoca, Romania; 7 Department of Endocrinology, “Carol Davila” University of Medicine and Pharmacy, Dionisie Lupu Street, Number 37, Sector 1, 020021 Bucharest, Romania; 8 Department of Clinical Endocrinology V, “C.I. Parhon” National Institute of Endocrinology, Aviatorilor Ave 34-36, Sector 1, 011863 Bucharest, Romania

**Keywords:** hormone, testosterone, leptin, chronic kidney disease, atherosclerosis

## Abstract

Chronic kidney disease (CKD) causes specific hormonal disturbances, such as variations in leptin and testosterone levels and function. These disturbances can promote errors in signaling interaction and cellular information processing and can be implicated in the pathogenesis of atherosclerosis. This study investigates the factors that affect leptin in CKD patients and examines how leptin is related to markers of vascular disease. We conducted a cross-sectional study of 162 patients with CKD in pre-dialysis and dialysis stages. We recorded clinical and laboratory data, including leptin, testosterone, and subclinical atherosclerosis markers like brachial–ankle pulse wave velocity (ba PWV) in pre-dialysis CKD patients and flow-mediated vasodilation (FMD) and nitroglycerin-mediated vasodilation (NMD) in hemodialysis (HD) patients. Leptin was significantly correlated with testosterone in CKD pre-dialysis stages (*p* < 0.001) and also in HD (*p* = 0.026), with adipose tissue mass in pre-dialysis stages (*p* < 0.001), and also in HD (*p* < 0.001). In women HD patients, leptin correlated with NMD (*p* = 0.039; r = −0.379); in all HD patients, leptin correlated with C reactive protein (*p* = 0.007; r = 0.28) and parathormone (*p* = 0.039; r = −0.220). Our research emphasizes the connection between leptin, adipose tissue, and testosterone in all stages of CKD. Leptin was associated with NMD in HD women and correlated with inflammatory syndrome and parathyroid hormone in all HD patients.

## 1. Introduction

Chronic kidney disease (CKD) leads to specific metabolic disorders, including hormonal disturbances and persistent inflammatory syndrome. These disorders increase the risk of atherosclerosis and cardiovascular disease to an extremely high level, which exceeds the risk in the general population, worsens the progression of CKD, and leads to high mortality rates. Hormonal disturbances, including modifications in leptin, ghrelin, testosterone, and prolactin levels, are commonly seen in CKD patients. In humans, circulating leptin comes primarily from visceral and subcutaneous adipose tissue [1] but can also be secreted by normal human osteoblasts, subchondral osteoblasts, placental syncytiotrophoblasts, and gastric epithelium [2,3]. In blood, leptin circulates in free and protein-bound forms; biologically active leptin exists in the free form. At the renal level, it is freely filtered glomerular; then, it is captured tubularly and degraded [4,5]. The basic mechanism of tubular reabsorption of leptin is receptor-mediated endocytosis [6]. Thus, it reaches the urinary level in a small amount.

The leptin receptor LepR is found in various tissues, such as adipose tissue, heart, muscle, lung, small intestine, liver, and the central nervous system, especially the hypothalamus [7]. Leptin is a hormone that plays a crucial role in regulating food intake [8]. Through neurohormonal mechanisms, increasing leptin can reduce food intake and improve insulin sensitivity. Leptin also helps in the maturation of the gonads, the regulation of the immune system, and mineral bone metabolism [8]. This hormone intervenes in metabolic regulation in several ways: (1) by significantly reducing appetite, it reduces energy intake [9,10], (2) it increases energy consumption by stimulating the activity of the sympathetic nervous system in the cardiovascular system or by increasing thermogenesis [11,12,13,14,15], (3) it increases lipolysis and the use of lipids as energy fuel [11,13], and (4) it increases glucose turnover and glucose absorption in brown adipose tissue, brain, and heart [16,17].

In addition to all the favorable effects of leptin mentioned above, some studies have indicated that its functions are altered in certain pathological conditions, possibly secondary to errors in intercellular signaling. Leptin can increase oxidative stress, promote endothelial dysfunction, and increase arterial stiffness [18]. As a result of these phenomena, the vulnerability of the atherosclerotic plaque and the risk of thrombosis increase [19]. Statins and antidiabetic drugs (including sitagliptin, liraglutide, and empagliflozin) can modify leptin levels [18]. In addition, leptin can stimulate the activity of the sympathetic nervous system, leading to hypertension [20]. The correlation of leptin with blood pressure values and adipose tissue mass brought into discussion a new possible therapeutic intervention for treating hypertension associated with obesity [21].

Leptin levels are high in CKD patients as an active and free form. However, not all CKD patients have high serum leptin levels, but after adjusting for adipose mass and age, hyperleptinemia was identified in all patients [5]. The high values of leptin in CKD are not solely due to kidney failure; other factors, such as inflammation, reduced erythropoietin levels, hyperinsulinemia, the type of dialysis, dialyzer membrane, and diet, may also be involved [2,22,23].

Hyperleptinemia in CKD has been shown to contribute to insulin resistance, protein-energy wasting, and atherogenic lipid profiles [24,25,26]. In peritoneal dialysis, it was associated with left ventricular hypertrophy [27]. In chronic hemodialysis (HD), low leptin levels have been associated with increased mortality [28] and a higher risk of cardiovascular events [29], and high leptin values were associated with vascular access dysfunction [30]. In patients with end-stage kidney disease, higher leptin values were recorded in those with a history of stroke. Still, among patients with stroke, serum leptin values were lower in those who also had congestive heart failure [18].

Thus, the role of leptin in CKD is not fully understood due to limited research and conflicting data. Therefore, further research is needed to clarify its role in CKD and its impact on cardiovascular disease. The purpose of this study is to evaluate the factors that influence the level of leptin in CKD patients in different stages and to determine the predictor of leptin levels. Additionally, the study aims to investigate the relationship between leptin levels and subclinical markers of atherosclerosis, such as nitroglycerin-mediated vasodilation (NMD), flow-mediated vasodilation (FMD), and pulse wave velocity (PWV) in CKD.

## 2. Results

The average age of patients in the pre-dialysis stage was 68 years, while in the HD group, it was 61.5 years. Among the patients receiving chronic HD, 56.8% were men, while in the pre-dialysis CKD group, 51.3% were men. Additionally, 19.5% of patients undergoing chronic HD and 37.8% of those with pre-dialytic CKD had diabetes. Table 1 shows the characteristics of the patients and the comparison between groups.

When comparing nutritional markers, leptin, and testosterone levels in men and women in both chronic HD patients and in pre-dialysis patients, we found that higher testosterone levels in men were associated with increased lean tissue mass (LTM), decreased adipose tissue mass (ATM) (only in HD group), and lower leptin levels (see Table 2).

### 2.1. Hemodialysis Group

The level of leptin in the group treated with HD was directly related to body mass index (BMI), ATM, the inflammatory syndrome indicated by white blood cells (WBCs) and high-sensitive C-reactive protein (hs-CRP), triglycerides, and low-density lipoprotein (LDL) cholesterol. It was inversely related to testosterone levels, intact parathyroid hormone (iPTH), and LTM. The multivariate analysis showed that leptin levels remained significantly associated with ATM, hs-CRP, and testosterone levels (see Table 3).

**Figure 1:**
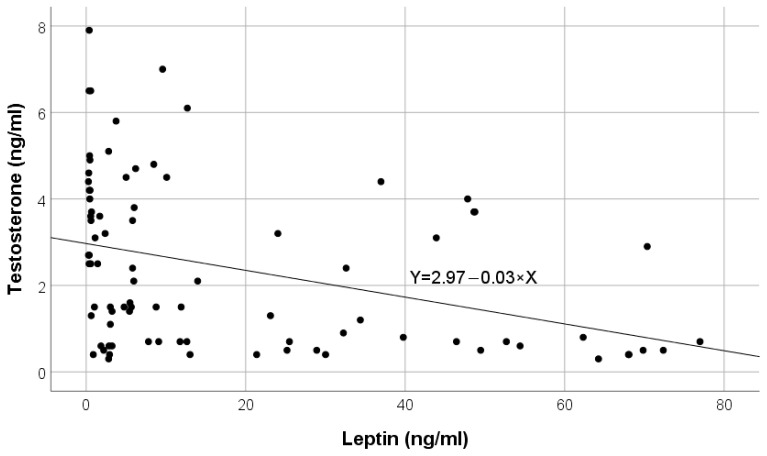
Variation in testosterone according to leptin in hemodialysis (HD) group A.

Figure 1 illustrates the inverse correlation between leptin values and testosterone in the HD group A.

**Figure 2:**
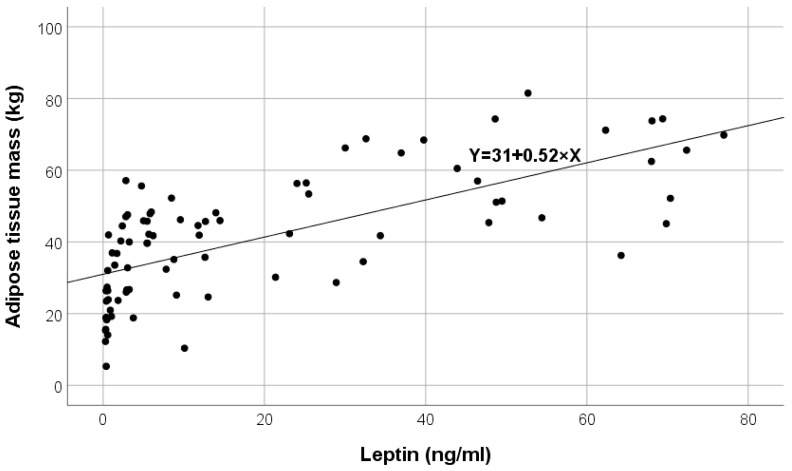
Variation in adipose tissue mass (ATM) according to leptin in hemodialysis (HD) group A.

The direct correlation between leptin values and ATM in the HD group A is shown in Figure 2.

Testosterone in HD group A was directly correlated with markers of protein metabolism such as pre-dialysis serum creatinine, serum phosphorus, and LTM but also with hemoglobin and diastolic blood pressure, and inversely with age, LDL cholesterol and total cholesterol, ferritin, and leptin. Multivariate analysis showed that testosterone remained significantly associated with hemoglobin and leptin. Interestingly, in the group of chronic HD men, testosterone correlated only with hemoglobin and NMD (see Table 4).

### 2.2. Pre-Dialysis Group

The leptin values in the pre-dialysis group correlated directly with BMI, ATM, serum calcium, serum bicarbonate, and eGFR, and inversely with testosterone levels, diastolic blood pressure (DBP), ferritin, and LTM. In the multivariate analysis, the leptin level remained significantly associated with ATM, BMI, LTM, and testosterone levels (see Table 5).

**Figure 3:**
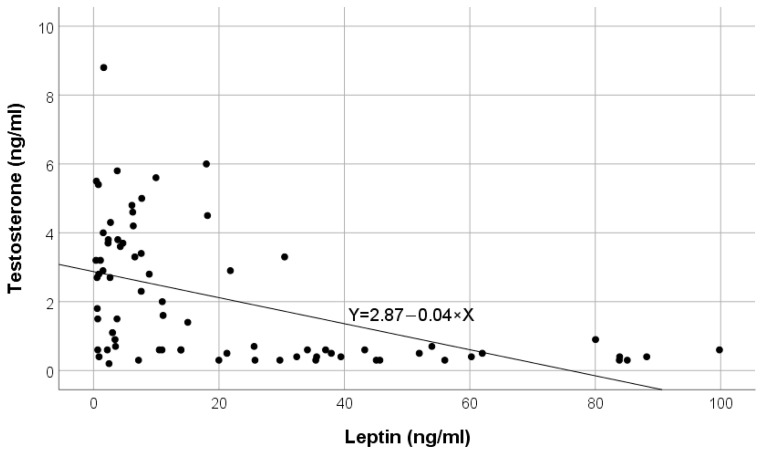
Variation in testosterone according to leptin in pre-dialysis chronic kidney disease (CKD) patients.

The inverse correlation of leptin values with testosterone in the pre-dialysis group is shown in Figure 3.

**Figure 4:**
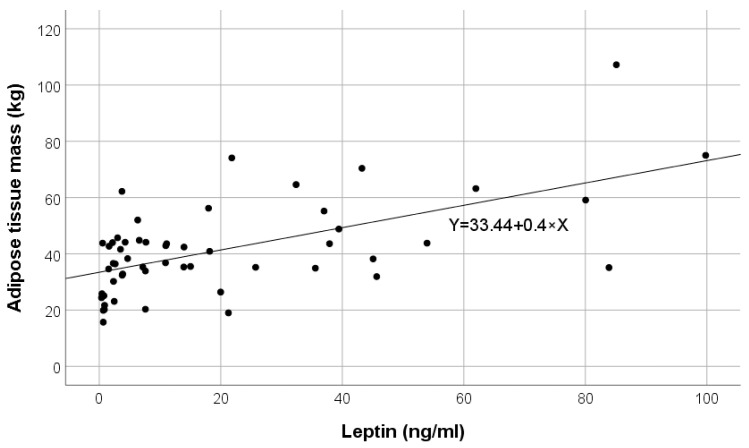
Variation in adipose tissue mass (ATM) according to leptin in pre-dialysis chronic kidney disease (CKD) patients.

Figure 4 shows the direct correlation of leptin values with ATM in the pre-dialysis group.

Testosterone in the pre-dialysis group was directly correlated with markers of protein metabolism, such as LTM, and inversely with leptin and calcium. Multivariate analysis showed that testosterone remained significantly associated with LTM and leptin. Interestingly, in the group of men in the pre-dialysis stages of CKD, testosterone was not associated with any of the monitored parameters (see Table 6).

## 3. Discussion

According to our study, leptin levels in all patients were significantly influenced by their adipose tissue mass and serum testosterone regardless of the CKD stage. This suggests that the main factor influencing leptin levels in the CKD patients studied is their body fat mass [31,32,33] not renal function. A higher value of adipose tissue mass was linked to higher levels of leptin in the blood, which is consistent with findings from other studies. The association of elevated leptin, an anorexigenic hormone, with high ATM values suggests the possibility of leptin resistance [34]. Adipocytes secrete leptin in CKD due to several mechanisms.

First, adipose tissue can lead to hyperinsulinism, which may increase serum leptin [35]. Second, the fatty acid profile specifically altered in CKD can increase leptin gene expression in subcutaneous adipose tissue and cause hyperleptinemia. The link between fatty acid disorders in CKD and the leptin gene also explains the relationship between leptin and lipids in our dialysis patients. Such correlations have been identified in CKD and the general population [26,36,37,38]. Thirdly, leptin mRNA is high in adipose tissue, which can cause increased serum leptin [39]. It is noted that visceral adipose tissue is particularly associated with hyperleptinemia in CKD [33], but in the general population, it was shown that leptin is mainly produced by subcutaneous white adipose tissue [40]. We mention a positive correlation between waist circumference, a marker of visceral adipose tissue, and leptin in our chronic HD patients.

High leptin levels associated with high adipose tissue mass may favor hypogonadism in the general population and chronic renal failure. This mechanism can explain our study’s inverse correlation between leptin and testosterone [39,41,42,43,44]. In fact, high leptin levels can increase estrogen levels, leading to increased aromatase activity. High aromatase activity can inhibit the hypothalamic–pituitary–gonadal axis and reduce testosterone levels [42]. In addition, in our study, HD women patients with higher ATM levels had higher leptin levels and lower muscle mass than males. The difference in circulating leptin levels between genders is known and may be explained by the higher proportion of adipose tissue and an increased production rate of leptin per unit mass of adipose tissue in women compared to men in the general population [45]. In the case of CKD patients, the findings are conflicting. Some studies show higher levels of leptin in women [46], while in diabetes patients with nephropathy, no gender-based variations in leptin levels were observed [47]. In addition, it was noted that the impact of leptin on weight regulation differs between genders [48]. Testosterone stimulates muscle mass formation by increasing muscle protein production and promoting myoblast differentiation [41]. In our study, muscle tissue mass was a common predictor of testosterone values in the pre-dialysis and dialysis stages. Interestingly, in men with chronic HD, the only marker associated with testosterone was hemoglobin, while in pre-dialysis, none of the analyzed parameters was correlated with serum testosterone. In normal physiology, leptin and testosterone levels may not have an inverse relationship. In situations where there is a mild to moderate leptin deficiency or in states of energy deficit such as fasting, the administration of leptin in a physiological dose can restore the level of androgenic and estrogenic hormones through its effect on the hypothalamic–pituitary–gonadal axis [1,49]. This means that low testosterone levels are associated with low leptin levels. After leptin administration, an increase in testosterone levels was also observed, indicating that the two hormones are directly correlated. These effects highlight the important role of leptin in neuroendocrine regulation in situations where there is a lack of energy and where the gonadal system is suppressed to conserve energy [50] and a lack of normal hormonal signaling in CKD patients.

It is also interesting to note that in our study, the mean testosterone level was found to be higher in the dialytic stage compared to the pre-dialysis stages, with no significant differences in the number of men, especially compared to the patients in stage 3 KDIGO (Kidney Disease Improving Global Outcomes) classification. In other studies, it is emphasized that the number of patients with hypogonadism and low testosterone increases with the progression of CKD [51]. However, several aspects were observed during the analysis of our study patients. First, in stage 3, KDIGO CKD patients were significantly older, and testosterone levels declined with age [52]. Second, stage 3 patients had more abundant ATM and higher serum leptin values, and we discussed the interrelationship between these parameters above.

Testosterone positively affects male cardiovascular health in a concentration-dependent manner in the general population [53], but studies on CKD are limited [54,55]. We found that elevated testosterone levels in male HD patients are associated with low NMD values, a marker of vascular smooth muscle dysfunction, and thus not with cardiovascular protection as in the general population. This association in men, which is somewhat inconsistent with data in the general population, could be related to differences in the cellular response to testosterone in CKD. Vascular endothelial cells contain receptors for androgen hormones, and their activation may vary by sex or other conditions [56].

In addition to the relationship of leptin with adipose tissue and serum testosterone, many other factors and multiple metabolic disorders may condition leptin levels in CKD patients. We observed that serum leptin was related to markers of the inflammatory syndrome and mineral and bone metabolism in the whole group. We recorded associations between leptin, eGFR, and blood glucose in pre-dialysis CKD patients.

In our chronic HD patients, we observed a significant association between leptin levels and hs-CRP as a marker of inflammatory syndrome. We found that patients with high hs-CRP and WBC values also had high leptin values, which is consistent with other studies’ findings [32]. It should be noted that the relationship between inflammatory syndrome and leptin is quite complex and warrants further analysis. On the one hand, studies have shown that in CKD, tumor necrosis factor (TNF) alpha can trigger the production and release of leptin from adipose tissue [57]. Simultaneously, other inflammatory molecules such as hs-CRP, interleukin (IL)-6, and IL-10 can also rise [31]. On the other hand, hyperleptinemia can induce the synthesis of inflammatory mediators and reactive oxygen species [58,59]. As the CKD severity rises, leptin, IL-6, and TNF alpha levels can also rise [60]. Some researchers believe leptin, TNF alpha, and IL-6 are pro-inflammatory cytokines activating the nuclear transcription factor kappa B. This activation can reduce protein synthesis and stimulate the ubiquitin-mediated proteolytic system, causing protein degradation [61]. In addition to the one mediated by testosterone, this mechanism would explain the association of high leptin values with reduced muscle mass in CKD patients.

The leptin levels in our study were directly correlated with factors such as ATM, triglycerides, increased glucose level, and markers of the inflammatory syndrome. These correlations suggest that hyperleptinemia in CKD is associated with an increase in the risk of atherosclerosis. It was mentioned that atherosclerosis is an indirect effect of high leptin levels [62]. Surrogate markers such as NMD, FMD, and PWV are used to detect subclinical atherosclerosis. In our study, we found that chronic HD women with high leptin values had low NMD values, indicating a relationship between hyperleptinemia and vascular smooth muscle dysfunction, which has not been noted in other studies. In the general population, the reduction in ATM was found to lead to a reduction in leptin levels associated with an improvement in endothelial function (increase in FMD) but without any effect on NMD [63]. Experimental data and clinical studies in CKD indicate that leptin in high concentrations can produce endothelial dysfunction by modifying the f-actin cytoskeleton through a mechanism involving the protein kinase B/glycogen synthase kinase β (AKT/GSK3β), nitric oxide, and β-catenin pathway [64,65]. We did not record associations of serum leptin with endothelial dysfunction, but we followed only FMD and not molecules such as vascular cell adhesion protein 1 (V-CAM 1) and intercellular adhesion molecule 1 (I-CAM 1), as in other studies [64].

Apart from these links to atherosclerosis, leptin, a hormone produced by adipose tissue and osteoblasts, plays a crucial role in mineral and bone metabolism. Studies have shown that leptin can stimulate osteoblasts, which are responsible for bone restoration [13], and inhibit osteoclast differentiation [66]. This positive effect on bones is supported by research showing that leptin is associated with reduced bone turnover and improved bone mineral density in patients with end-stage renal disease [67]. In chronic HD patients, leptin levels, serum albumin, and body weight correlate positively with bone mineral density [63,68]. In the same way, our findings in chronic HD patients showed an inverse correlation between leptin and iPTH, which is a hormone that can increase bone turnover and reduce bone mineral density. However, high leptin levels in CKD are not always associated with positive effects on bone. Bone resistance to leptin in CKD has been discussed due to leptin’s two modes of action: a direct stimulatory effect on bone and an opposite indirect effect via the central nervous system [66].

In the dialytic stage, inflammatory syndrome markers and mineral and bone metabolism markers, such as iPTH, significantly correlated with the leptin level. The factors associated with serum leptin in CKD in pre-dialysis stages are similar to those identified in the general population.

Leptin, a glomerular filtered and tubularly metabolized hormone, may increase in CKD patients as eGFR decreases, as shown in other studies [69,70]. In contrast, our research found that leptin levels increased with increasing eGFR and in parallel with increasing ATM. The difference between our study and other results is that we did not adjust leptin levels for age or ATM [70]. Renal function has been observed to only partially influence leptin levels in CKD patients, the main determinant being leptin overproduction in adipose tissue due to hyperinsulinemia and chronic inflammation [35,71]. In turn, leptin can influence kidney function. It can stimulate the synthesis of TGF beta, a molecule with a fibrogenesis role, causing the progression of CKD [72]. Hyperleptinemia may predict a decline in renal function in men over time regardless of the presence of diabetes or hypertension [73].

This study is significant because it highlights the connections of leptin in all phases of CKD and reveals relationships that have not been emphasized in other research. Specifically, it highlights the strong correlation of leptin with ATM and testosterone in pre-dialysis and dialysis patients, the gender variation in leptin in CKD, and the association of leptin with vascular smooth muscle dysfunction as an atherosclerosis marker in women undergoing chronic HD. The multiple connections of leptin in CKD indicate that it intersects with multiple metabolic pathways and pathogenic cascades. Future research will determine whether leptin can be a potential therapeutic target in CKD patients.

Our study has several limitations. First, it included a relatively small number of patients in each stage of CKD, so further studies are needed to confirm the interactions of leptin in this population group. Second, the study was observational by design, so the findings need to be confirmed in a prospective study. Third, there was no control group. Additionally, due to the nature of our cross-sectional data, this study was limited in interpreting causality.

## 4. Materials and Methods

### 4.1. Participants

We conducted a cross-sectional observational study involving 162 patients with CKD: 88 patients were in chronic HD treatment in Nefromed Dialysis Center Cluj-Napoca, and 74 patients were in the pre-dialysis stages of CKD in the monitorization of Cluj County Emergency Clinical Hospital Department of Nephrology. All included patients met the inclusion and exclusion criteria and signed informed consent. All procedures in the study followed institutional and national research committee ethical standards and the 1964 Declaration of Helsinki and its subsequent amendments. The Ethics Committee of “Iuliu Hatieganu” University of Medicine and Pharmacy Cluj-Napoca approved the study with IRB number 348/26 September 2017.

The inclusion criteria for pre-dialysis patients were age ≥ 18 years, diagnosis of CKD stage 3–5 pre-dialysis, defined according to the KDIGO [74], and no kidney transplant for at least six months, having stable renal function in the last three months (<5 mL/min/1.73 m^2^ change in eGFR). The inclusion criteria for HD patients were prevalent HD patients, age > 18 years, and duration of HD at least 6 months (HD vintage). All patients were on a thrice-weekly HD (4–5 h) regimen.

Exclusion criteria for all patients were the following: acute inflammation, severe neoplasia with a life expectancy of <6 months, hepatitis virus infection, and any other serious chronic or acute diseases requiring treatment or absence of data.

### 4.2. Methods

Demographic data, comorbidities (diabetes, hypertension), and medication at enrollment were obtained from medical records. We also registered clinical data: age, weight, height, systolic blood pressure (SBP), and DBP. Hypertension was diagnosed according to SBP/DBP ≥ 140/90 mmHg, as well as the use of relevant medications. We registered laboratory data (such as urea, creatinine, sodium, potassium, calcium, phosphorus, total cholesterol, LDL-cholesterol, high-density lipoprotein (HDL) cholesterol, blood glucose, iPTH, hs-CRP, hemoglobin, WBC count, calcium, phosphorus, ferritin, albumin, alkaline phosphatase, serum bicarbonate, and serum creatinine in the pre-dialysis stage). Additionally, we have determined the levels of leptin, free testosterone, and prolactin using ELISA, with a minimum detection limit of 0.15 ng/mL for leptin and 0.06 pg./mL for testosterone.

We calculated pulse pressure (PP), PP = SBP − DBP, and BMI based on the formula BMI = weight (kg)/[height (m^2^)]. Nutrition was also assessed by bioimpedance using the Body Composition Monitor, which is a certified device (manufactured by Fresenius Medical Care, Bad Homburg, Germany) that showed LTM (kg) and ATM (kg) and also calculated lean and fat tissue indexes (LTI and FTI, respectively) [75].

In addition, we measured vascular function in HD patients using markers such as NMD for vascular smooth muscle function and FMD for endothelial function. NMD and FMD were evaluated using high-resolution ultrasound: GE Logiq3 (General Electric Company, Fairfield, CT, USA). In pre-dialysis CKD patients, we evaluated the arterial stiffness expressed by PWV with the Mobil-O-Graph NG device (Medexpert Ltd., Budapest, Hungary).

Our published articles [76,77] described the procedure for measuring NMD, FMD, and PWV.

### 4.3. Statistical Analysis

The data were presented using the following parameters: qualitative data with absolute and relative frequencies; normally distributed quantitative data with averages and standard deviations; non-normally distributed quantitative data with medians, the 25th, and the 75th percentiles.

Data analysis procedures included conducting an ANOVA test (if normal distribution and variance equality were present) or a Kruskal–Wallis test for the comparison of four means, followed by post-doc analysis, with the use of Scheffe post-doc analysis for ANOVA and the Bonferroni correction for Kruskal–Wallis. A Chi-square test was conducted to compare four frequencies, which was followed by Bonferroni correction. For correlation analysis, the Pearson correlation coefficient was calculated for normally distributed data, and the Spearman correlation coefficient was calculated for non-normally distributed data.

Only significant or almost significant correlations were presented in tables. All the variables significantly or almost significantly correlated with leptin or testosterone were considered as independent variables for multivariate analysis. In multivariate linear analysis, the dependent variables were leptin and testosterone. Only the significant variables in the multivariate models were reported in the tables.

IBM SPSS Statistics for Windows, Version 25.0. IBM Corp., Armonk, NY, USA was used for the analysis. The significance threshold of 0.05 was taken.

## 5. Conclusions

Our study shows the connection between leptin, adipose tissue, and testosterone in all stages of CKD. We also note that hyperleptinemia in our CKD patients was linked to other proatherogenic factors, such as dyslipidemia and hyperglycemia in all CKD stages and with vascular smooth muscle dysfunction in chronic hemodialyzed women. Additionally, we observe the gender variation in leptin in CKD: in advanced stages of CKD, the inflammatory syndrome impacts leptin values, and leptin is associated with parathormone.

## Figures and Tables

**Table 1:** Comparisons between groups according to estimated glomerular filtration rate (eGFR) and dialysis treatment.

Variable	Group A Hemodialysis (n = 88)	Group B Std. V KDIGO (n = 23)	Group C Std. IV KDIGO (n = 26)	Group D Std. III KDIGO (n = 25)	*p*
Male sex n (%)	50 (56.8)	12 (52.2)	12 (46.2)	14 (56)	0.803
Age (years)	61.5 (54, 71) ^b,c^	65 (60, 70)	69 (62, 78)	70 (65, 75)	**0.003**
WC (cm)	98 (84, 108)	104 (93.5, 111)	96 (90, 106)	105 (104, 108)	0.094
BMI (kg/m^2^)	27.54 (23.2, 31)	27.3 (25.6, 29.9)	28.25 (24.85, 29.6)	29.1 (27.7, 33.5)	0.133
LTM (kg)	29.71 (25.2, 37.64)	33.9 (29.25, 52.65)	39.5 (26.25, 45)	35.35 (26.6, 42.45)	0.065
ATM (kg)	41.75 (26.51, 51.38)	35.3 (23.75, 38.75) ^e^	35.5 (32.15, 43.7) ^f^	46.8 (40.45, 60.65)	**0.009**
SBP (mmHg)	142.32 ± 21.29	155.76 ± 24.48	145.81 ± 23.88	144.67 ± 18.21	0.140
DBP (mmHg)	75 (67, 80) ^a,b,c^	94 (86, 98)	80 (74, 92)	82 (80, 98)	**<0.001**
PP (mmHg)	68.28 ± 18.77	62.18 ± 18.06	62.33 ± 20.16	58.67 ± 15.41	0.115
Diabetes n (%)	17 (19.5) ^a,b,c^	10 (43.5)	10 (38.5)	8 (32)	0.059
Hypertension n (%)	62 (70.5) ^b^	18 (85.7)	24 (100)	19 (90.5)	**0.005**
Fasting glucose (mg/dL)	93.89 (87.43, 113)	99 (91, 129.5)	108 (90, 139.5)	101 (94, 122)	0.180
Triglycerides (mg/dL)	134 (95.33, 181.16)	131 (99.5, 170.5)	148 (116, 188)	126 (93, 159.5)	0.675
LDL-cholesterol (mg/dL)	100.21 ± 36.88	120.53 ± 51.74	123.35 ± 46.66	127.84 ± 38.3	**0.015**
Total cholesterol (mg/dL)	173.15 ± 40.6	173.89 ± 39.16	173.96 ± 32.66	188.33 ± 35.58	0.386
HDL-cholesterol (mg/dL)	39.61 (30.75, 47.64)	41 (32.5, 47.5)	35.5 (28, 41)	45 (39, 49.5)	0.111
Hemoglobin (g/dL)	11.5 (10.65, 12.3) ^c^	11 (10.3, 11.8) ^e^	12.05 (10.3, 13.2)	13.4 (12, 14)	**0.001**
Ferritin (ng/mL)	567 (333.03, 782.65) ^a,b,c^	178.5 (63.5, 351)	98 (34, 186)	88.5 (61.5, 157)	**<0.001**
Hs-CRP (mg/dL)	0.59 (0.23, 1.33)	0.5 (0.2, 1.27)	0.53 (0.29, 1.19)	0.38 (0.23, 0.89)	0.660
WBC (no./mmc)	6440 (5530, 7815) ^c^	7770 (6120, 9800)	7315 (6200, 8370)	8630 (6500, 8930)	**0.005**
Bicarbonate level (mEq/L)	21.4 (18.45, 24.25)	19.15 (16.3, 20.85)	19.9 (17.3, 23.6)	20 (17.7, 22.3)	0.068
Calcium (mg/dL)	8.9 (8.32, 9.2)	8.66 (8.04, 9.1) ^c^	9.23 (8.64, 9.68)	9.32 (8.94, 9.62)	**0.003**
Phosphorus (mg/dL)	4.71 (3.98, 5.89) ^b,c^	4.72 (4.18, 5.81) ^d,e^	3.7 (3.16, 4.48)	3.15 (2.91, 3.53)	**<0.001**
AP (UI/L)	73 (55.92, 95.43)	86 (70, 98)	83 (77.5, 107.5)	75 (70, 110.5)	0.175
iPTH (pg/mL)	286.75 (164.65, 729.65) ^b,c^	283.95 (151.85, 398.85) ^d,e^	104.1 (52.15, 153.1)	104.2 (78.3, 152.45)	**<0.001**
Creatinine (mg/dL)	8.59 (7.3, 10.4) ^a,b,c^	4.7 (4.04, 6.33) ^d,e^	2.4 (2.09, 2.51) ^f^	1.56 (1.37, 1.68)	**<0.001**
Albumin (g/dL)	3.91 (3.7, 4.08) ^b,c^	3.76 (3.56, 4.13) ^d^	3.57 (3.47, 3.82) ^f^	4.38 (3.9, 4.46)	**0.002**
eGFR (mL/min/m^2^)		10.17 ± 3.11 ^d,e^	23.63 ± 4.3 ^f^	41.68 ± 8.13	**<0.001**
Testosterone (ng/mL)	2.1 (0.7, 4)	2.7 (0.6, 3.55)	0.65 (0.4, 3.6)	0.9 (0.4, 3.2)	0.076
Leptin (ng/mL)	5.99 (1.57, 31.14)	3.41 (1.9, 9.67) ^e^	14.48 (3.86, 37.9)	19.98 (6.33, 39.44)	**0.020**
Betablockers n (%)	50 (56.8)	12 (54.5)	13 (52)	14 (66.7)	0.774
ACEI + ARB	37 (42)	7 (31.8)	14 (56)	11 (52.4)	0.319
Statin n (%)	16 (18.2)	8 (42.1)	6 (24)	7 (33.3)	0.111
Antiagregants n (%)	32 (36.4)	4 (19)	10 (40)	5 (23.8)	0.301
Ba PWV (cm/s)	-	9.98 ± 2.5	11.12 ± 2.25	10.65 ± 2.11	0.324
NMD (%)	7.25 (2.27, 12.5)	-	-	-	-
FMD (%)	8.33 (4.31, 14.29)	-	-	-	-

^a^ *p* < 0.05 when comparing group a with group b; ^b^ *p* < 0.05 when comparing group a with group c; ^c^ *p* < 0.05 when comparing group a with group d; ^d^ *p* < 0.05 when comparing group b with group c; ^e^ *p* < 0.05 when comparing group b with group d; ^f^ *p* < 0.05 when comparing group c with group d; significant *p* value with bold; std.—stadium; n—number of people; no.—number of cells; BMI—body mass index; SBP—systolic blood pressure; DBP—diastolic blood pressure; PP—pulse pressure; eGFR—estimated glomerular filtration rate; LTM—lean tissue mass; ATM—adipose tissue mass; LDL—low-density lipoprotein; HDL—high-density lipoprotein; iPTH—Intact parathyroid hormone; Ba PWV—brachial–ankle pulse wave velocity; ACEI—angiotensin-converting enzyme inhibitors; ARB—angiotensin II receptor blockers; WC—waist circumference; NMD—nitroglycerin-mediated vasodilatation; FMD—flow-mediated vasodilation; hs-CRP—high-sensitive C-reactive protein; AP—alkaline phosphatase; WBC—white blood cell; arithmetic mean ± standard deviation; median (25th–75th percentile).

**Table 2:** Comparisons between men and women in group A with dialysis treatment and in pre-dialysis group.

Variable	Group A Hemodialysis (n = 88)			Group Pre-Dialysis (n = 74)		
	Women (n = 38)	Men (n = 50)	*p*	Women (n = 36)	Men (n = 38)	*p*
Age (years)	66 (56, 75)	59 (52, 68)	**0.032**	70 (65, 77)	67 (61, 71)	0.122
WC (cm)	99 (80, 115)	97.5 (85, 104)	0.622	105 (91, 107)	104 (97, 109)	0.254
BMI (kg/m^2^)	29.12 (24.38, 36.48)	26.54 (22.83, 29.53)	**0.010**	29.85 (26.4, 33.3)	28.2 (26.75, 29.1)	0.168
LTM (kg)	25.98 (22.47, 27.96)	35.56 (30.12, 43.41)	**<0.001**	27.1 (24.7, 34.7)	42.4 (36, 52.7)	**<0.001**
ATM (kg)	43.44 (30.15, 62.45)	39.65 (24.95, 46.62)	**0.013**	40.9 (35.1, 55.2)	36.6 (30.2, 44.1)	0.167
SBP (mmHg)	139.26 ± 18.81	144.64 ± 22.92	0.243	143 (127, 162)	151 (130, 170)	0.254
DBP (mmHg)	70 (67, 78)	80 (70, 82)	**0.020**	80.5 (74, 96)	91 (81, 99)	**0.022**
PP (mmHg)	68.66 ± 19.23	68 ± 18.6	0.872	66 (44, 74)	57 (50, 72)	0.856
Diabetes n (%)	6 (15.8)	11 (22.4)	0.437	13 (36.1)	15 (39.5)	0.766
Hypertension n (%)	26 (68.4)	36 (72)	0.715	26 (86.7)	35 (97.2)	0.169
Fasting glucose (mg/dL)	93.5 (87.2, 110)	94 (87.65, 115)	0.150	108.5 (99, 139)	97 (89, 124.5)	0.896
Triglycerides (mg/dL)	145.97 (102, 225)	125.69 (94.05, 163.51)	0.080	148 (114.5, 171.5)	126 (93, 155.5)	0.541
LDL-cholesterol (mg/dL)	106 ± 36.3	95.81 ± 37.06	0.201	106.8 (76.2, 140)	136 (105, 163)	0.055
Total cholesterol (mg/dL)	184.89 ± 40.36	164.22 ± 38.83	**0.017**	171.5 (153.5, 201)	176 (155, 195.5)	0.754
HDL-cholesterol (mg/dL)	41.25 (30.14, 49)	36.5 (31.69, 45.8)	0.374	42 (32.5, 48.5)	39 (33, 46)	0.778
Hemoglobin (g/dL)	11.05 (10.6, 11.6)	12 (10.8, 12.6)	**0.015**	11.55 (10.75, 12.5)	13 (10.3, 14.8)	0.306
Ferritin (ng/mL)	665.7 (402.34, 846.6)	472 (317.5, 723)	**0.033**	91 (55, 186)	106 (63, 279.5)	0.679
Hs-CRP (mg/dL)	0.61 (0.34, 1.43)	0.53 (0.21, 1.32)	0.440	0.43 (0.2, 0.91)	0.52 (0.34, 1.42)	0.236
WBC (no./mmc)	6210 (5530, 7440)	6495 (5260, 7880)	0.947	7065 (6160, 8875)	7800 (6410, 9060)	0.146
Bicarbonate level (mEq/L)	22.8 (18.2, 24.1)	20.5 (18.6, 24.5)	0.330	19 (17.3, 21.1)	19.6 (16.5, 22.3)	0.673
Calcium (mg/dL)	8.97 (8.4, 9.2)	8.79 (8.24, 9.2)	0.303	9.3 (8.93, 9.68)	8.79 (8.44, 9.3)	0.314
Phosphorus (mg/dL)	4.5 (3.8, 5.19)	5.12 (4.08, 6.84)	0.127	4.08 (3.48, 4.88)	3.56 (2.93, 4.5)	0.249
AP (UI/L)	79.61 (61, 100)	66.2 (51.83, 94.44)	0.130	89.5 (78, 106)	77 (68, 105.5)	0.095
iPTH (pg/mL)	266.5 (158.7, 481.85)	336 (185.7, 798)	0.179	140 (94.35, 260.95)	118.4 (87.5, 202.6)	0.176
Creatinine (mg/dL)	8.1 (6.8, 8.73)	9.42 (7.7, 11.3)	**<0.001**	2.18 (1.6, 3.44)	2.47 (1.8, 4.5)	0.225
Albumin (g/dL)	3.87 (3.62, 4)	3.94 (3.74, 4.13)	0.053	3.79 (3.61, 4.07)	3.76 (3.49, 4.26)	0.875
eGFR (mL/min/m^2^)				23 (13.5, 32)	26 (12, 38)	0.354
Testosterone (ng/mL)	0.7 (0.5, 1.3)	3.65 (2.5, 4.8)	**<0.001**	0.5 (0.3, 0.6)	3.3 (2.3, 4.3)	**<0.001**
Leptin (ng/mL)	24.3 (5.43, 49.47)	3.49 (0.57, 9.58)	**<0.001**	35.5 (13.93, 54.97)	3.82 (1.54, 7.68)	**<0.001**
Betablockers n (%)	21 (55.3)	29 (58)	0.797	19 (63.3)	20 (52.6)	0.376
ACEI + ARB	14 (36.8)	23 (46)	0.804	15 (50)	17 (44.7)	0.666
Statin n (%)	7 (18.4)	9 (18)	**0.002**	10 (35.7)	11 (29.7)	0.609
Antiagregants n (%)	17 (44.7)	15 (30)	0.155	7 (24.1)	12 (31.6)	0.503
Ba PWV (cm/s)				10.6 (9.7, 12.2)	10.65 (9.05, 11.95)	0.516
NMD (%)	9.68 (4.65, 14.09)	4.34 (1.83, 11.69)	**0.014**			
FMD (%)	11.11 (4.55, 15.38)	8.16 (4.08, 14.29)	0.454			
HD duration	69 (34, 88)	54 (22, 83)	0.034			

Significant *p* value with bold; n—number of people; no.—number of cells; BMI—body mass index; SBP—systolic blood pressure; DBP—diastolic blood pressure; PP—pulse pressure; eGFR—estimated glomerular filtration rate; LTM—lean tissue mass; ATM—adipose tissue mass; LDL—low-density lipoprotein; HDL—high-density lipoprotein; iPTH—intact parathyroid hormone; Ba PWV—brachial–ankle pulse wave velocity; ACEI—angiotensin-converting enzyme inhibitors; ARB—angiotensin II receptor blockers; WC—waist circumference; NMD—nitroglycerin-mediated vasodilatation; FMD—flow-mediated vasodilation; hs-CRP—high-sensitive C-reactive protein; AP—alkaline phosphatase; WBC—white blood cell; HD—hemodialysis; arithmetic mean ± standard deviation; median (25th–75th percentile).

**Table 3:** Correlation and multivariate linear regression between the parameters and leptin in hemodialysis (HD) group A.

Variable	Women in Group A (n = 38)		Men in Group A (n = 50)		Group A		Multivariate Analysis in Group A	
Leptin (ng/mL)	Correlation Coefficient	*p*	Correlation Coefficient	*p*	Correlation Coefficient	*p*	B Coefficient 95%CI (Lower, Upper)	*p*
Age (years)	0.166	0.320	0.159	0.269	0.210	**0.049**		
WC (cm)	0.624	**<0.001**	0.620	**<0.001**	0.558	**<0.001**		
DBP (mmHg)	−0.048	0.773	−0.168	0.243	−0.184	0.086		
Triglycerides (mg/dL)	0.152	0.361	0.201	0.162	0.235	**0.028**		
LDL-cholesterol (mg/dL)	0.004	0.983	0.271	0.057	0.221	**0.038**		
Total Cholesterol (mg/dL)	−0.056	0.740	0.367	**0.009**	0.270	**0.011**		
iPTH (pg/mL)	−0.122	0.464	−0.236	0.098	−0.220	**0.039**		
hs-CRP (mg/dL)	0.193	0.246	0.337	**0.017**	0.284	**0.007**	0.35 (0.07, 0.63)	**0.016**
WBC (no/mmc)	0.104	0.534	0.444	**0.001**	0.253	**0.017**		
Testosterone (ng/mL)	−0.153	0.359	0.230	0.108	−0.377	**<0.001**	−1.83 (−3.44, −0.23)	**0.026**
BMI (kg/m^2^)	0.713	**<0.001**	0.713	**<0.001**	0.737	**<0.001**		
LTM (kg)	−0.262	0.112	−0.271	0.065	−0.439	**<0.001**		
ATM (kg)	0.716	**<0.001**	0.787	**<0.001**	0.751	**<0.001**	0.86 (0.61, 1.12)	**<0.001**
NMD (%)	−0.379	**0.039**	−0.137	0.392	−0.177	0.139		

Significant *p* value with bold; n—number of people; BMI—body mass index; DBP—diastolic blood pressure; LTM—lean tissue mass; ATM—adipose tissue mass; LDL—low-density lipoprotein; iPTH—intact parathormone; WC—waist circumference; NMD—nitroglycerin-mediated vasodilatation; FMD—flow-mediated vasodilation; hs-CRP—high-sensitive C-reactive protein, WBC—white blood cell.

**Table 4:** Correlation between the parameters and testosterone in hemodialysis (HD) group A.

Variable	Women in Group A (n = 38)		Men in Group A (n = 50)		Group A		Multivariate Analysis of Group A	
Testosterone (ng/mL)	Correlation Coefficient	*p*	Correlation Coefficient	*p*	Correlation Coefficient	*p*	Correlation Coefficient	*p*
Age (years)	−0.119	0.475	−0.080	0.580	−0.221	**0.039**		
SBP (mmHg)	−0.150	0.369	0.185	0.199	0.179	0.095		
DBP (mmHg)	0.076	0.651	0.212	0.138	0.298	**0.005**		
Creatinine (mg/dL)	0.022	0.894	−0.079	0.585	0.269	**0.011**		
LDL cholesterol (mg/dL)	0.103	0.539	−0.142	0.324	−0.182	0.090		
Total cholesterol (mg/dL)	0.154	0.357	−0.170	0.238	−0.235	**0.027**		
Phosphorus (mg/dL)	0.279	0.090	0.140	0.331	0.223	**0.037**		
Ferritin (ng/mL)	0.149	0.371	−0.225	0.116	−0.236	**0.027**		
Hemoglobin (g/dL)	0.174	0.295	0.330	**0.019**	0.256	**0.016**	0.84 (0.38, 1.30)	**<0.001**
Leptin (ng/mL)	−0.153	0.359	0.230	0.108	−0.377	**<0.001**		
BMI (kg/m^2^)	−0.032	0.850	0.231	0.114	−0.200	0.064		
LTM (kg)	−0.147	0.378	0.145	0.332	0.510	**<0.001**	0.09 (0.03, 0.15)	**0.006**
NMD (%)	0.199	0.291	−0.316	**0.044**	−0.145	0.226		

Significant *p* value with bold; n—number of people; SBP—systolic blood pressure; DBP—diastolic blood pressure; LDL—low-density lipoprotein; BMI—body mass index; LTM—lean tissue mass; NMD—nitroglycerin-mediated vasodilatation.

**Table 5:** Correlation between the parameters and leptin in the pre-dialysis chronic kidney disease (CKD) patients (n = 73).

Variable	Women in Group Pre-Dialysis (n = 36)		Men in Group Pre-Dialysis (n = 38)		Group Pre-Dialysis		Multivariate Analysis on Group Pre-Dialysis	
Leptin (ng/mL)	Correlation Coefficient	*p*	Correlation Coefficient	*p*	Correlation Coefficient	*p*	B Coefficient 95%CI (Lower, Upper)	*p*
Testosterone (ng/mL)	−0.238	0.162	−0.113	0.499	−0.570	**<0.001**	−6.09 (−8.87, −3.30)	**<0.001**
DBP (mmHg)	−0.191	0.349	−0.205	0.253	−0.299	**0.021**	-	
Triglycerides (mg/dL)	0.426	0.061	0.065	0.748	0.254	0.084	-	
Calcium (mg/dL)	0.277	0.119	0.166	0.327	0.364	**0.002**	-	
Phosphorus (mg/dL)	−0.181	0.329	−0.317	0.064	−0.078	0.533		
iPTH (pg/mL)	−0.425	**0.027**	−0.109	0.545	−0.173	0.185		
Fasting glucose (mg/dL)	0.522	**0.009**	0.293	0.083	0.302	**0.019**	0.16 (0.04, 0.03)	**0.013**
Ferritin (ng/mL)	−0.169	0.453	−0.323	0.076	−0.294	**0.033**	-	
Bicarbonate level (mEq/L)	0.383	0.308	0.538	**0.021**	0.408	**0.034**	-	
BMI (kg/m^2^)	0.709	**<0.001**	0.505	**0.003**	0.595	**<0.001**	4.56 (3.02, 6.10)	**<0.001**
LTM (kg)	0.048	0.818	−0.094	0.628	−0.384	**0.004**	−0.97 (−1.4, −0.54)	**<0.001**
ATM (kg)	0.685	**<0.001**	0.446	**0.015**	0.591	**<0.001**	0.81 (0.48, 1.15)	**<0.001**
eGFR (mL/min/1.73 m^2^)	0.416	**0.012**	0.340	**0.037**	0.264	**0.023**	-	

Significant *p* value with bold; n—number of people; DBP—diastolic blood pressure; LDL—low-density lipoprotein; eGFR—estimated glomerular filtration rate; iPTH—intact parathyroid hormone; BMI—body mass index; LTM—lean tissue mass; ATM—adipose tissue mass.

**Table 6:** Correlation between the parameters and testosterone in pre-dialysis chronic kidney disease (CKD) patients.

Variable	Women in Group Pre-Dialysis (n = 36)		Men in Group Pre-Dialysis (n = 38)		Group Pre-Dialysis		Multivariate Analysis on Group Pre-Dialysis	
Testosterone (ng/mL)	Correlation Coefficient	*p*	Correlation Coefficient	*p*	Correlation Coefficient	*p*	B Coefficient 95%CI (Lower, Upper)	*p*
Leptin (ng/mL)	−0.238	0.162	−0.113	0.499	−0.570	**<0.001**	−0.03 (−0.05, −0.01)	**0.009**
WC (cm)	0.573	**0.040**	0.027	0.913	0.233	0.200		
SBP (mmHg)	−0.228	0.262	0.124	0.491	0.255	0.051		
LDL-cholesterol (mg/dL)	0.154	0.529	0.086	0.670	0.264	0.076		
Calcium (mg/dL)	−0.173	0.335	−0.056	0.743	−0.296	**0.013**		
LTM (kg)	0.227	0.275	0.022	0.911	0.450	**0.001**	0.05 (0.01, 0.09)	**0.029**
eGFR (mL/min/1.73 m^2^)	−0.336	**0.045**	0.054	0.746	−0.065	0.585		

Significant *p* value with bold; n—number of people; WC— waist circumference; SBP—sistolic blood pressure; LDL—low-density lipoprotein; eGFR—estimated glomerular filtration rate; LTM—lean tissue mass.

## Data Availability

The research data supporting this study’s findings are not publicly available. Further inquiries can be directed to the corresponding author.
